# Needs, Experiences, and Views of People With Rheumatic and Musculoskeletal Diseases on Self-Management Mobile Health Apps: Mixed Methods Study

**DOI:** 10.2196/14351

**Published:** 2020-04-20

**Authors:** Aurelie Najm, Heidi Lempp, Laure Gossec, Francis Berenbaum, Elena Nikiphorou

**Affiliations:** 1 Department of Rheumatology, Nantes University Hospital Nantes France; 2 INSERM UMR 1238 Nantes France; 3 Centre for Rheumatic Diseases, Department of Inflammation Biology, School of Immunology and Microbial Sciences, Faculty of Life Sciences & Medicine, King’s College London London United Kingdom; 4 Sorbonne Université, Institut Pierre Louis d'Epidémiologie et de Santé Publique INSERM UMR S1136 Paris France; 5 Rheumatology department, AP-HP Pitié Salpêtrière hospital Paris France; 6 Department of Rheumatology, AP-HP, Saint Antoine Hospital Paris France; 7 Sorbonne Université and INSERM CRSA Paris France

**Keywords:** mHealth, mixed methods, mobile health apps, rheumatic and musculoskeletal disease, smartphone, apps, rheumatoid arthritis, digital health, mobile health

## Abstract

**Background:**

Despite the growing interest and exponential popularity of mobile health (mHealth) apps for long-term conditions such as rheumatic and musculoskeletal diseases (RMDs) and their self-management, patients are rarely directly consulted and involved in the app development process.

**Objective:**

This study aims to explore the needs, experiences, and views of people diagnosed with RMDs on mHealth apps.

**Methods:**

The study used a mixed methods approach: (1) an initial qualitative phase via a patient focus group in the UK and (2) a survey disseminated through national organizations for patients with RMDs across European countries, the United States, Canada, and Australia.

**Results:**

The focus group included six patients with life-long musculoskeletal conditions. Half had used a self-management app at least once. The use of existing apps was reported as time-consuming due to a lack of functionality. The need for bespoke apps was voiced by all participants. Among 424 patients across European countries, the United States, Canada, and Australia, the main age group was 45 to 54 years (122/424, 28.7%), and 86.8% (368/424) were women. Half of the respondents were aware of the existence of apps to support self-management of their RMDs (188/355, 53%), with 42% (79/188) of them currently using such devices. Patients were mostly interested in an app to self-monitor their health parameters (259/346, 74.9%) and disease activity (221/346, 63.9%) or communicate directly with their health care provider (200/346, 57.8%).

**Conclusions:**

Patients considered that using an app could help them to self-manage their RMD condition if it was tailored to their needs and co-developed with health professionals. The development of such apps will require standardization and regular quality control.

## Introduction

The role of self-management in rheumatic and musculoskeletal diseases (RMDs) has become increasingly recognized, especially in support of holistic and patient-centered care [1]. A growing number of patients seek out various sources of information to better understand and manage their disease as part of their quest to take a more active role in their care [2,3]. Self-management refers to “the individual’s ability to manage the symptoms, treatment, physical and psychosocial consequences, and lifestyle changes inherent in living with a chronic condition” [4]. In line with this, the past decade has witnessed an exponential growth of mobile phones and other electronic apps. A cumulative willingness of healthy individuals to improve their lifestyle has led to the demand for new digital lifestyle management solutions. The use of apps could support people taking personal responsibility for their well-being and contribute to their disease management in a more proactive way. Indeed, the number of health-related devices downloaded from various app stores has more than doubled from 1.7 billion in 2013 to 3.7 billion in 2017 [5].

Most apps on the market cater to healthy individuals, with a focus on physical activity and diet [6]. In parallel, other electronic devices tailored to persons living with long-term conditions have emerged. Their potential as disease management tools has been described in various conditions, such as asthma [7], hypertension [8], diabetes mellitus [9], and other life-long conditions [10]. Moreover, mHealth apps can support self-management in long-term conditions, such as RMDs, but can also help empower individuals to take a more active role in their health and be part of shared decision making [11-13] between the patient and health care staff.

The existence of apps may enable patients who live with long-term physical or mental conditions to improve their self-management. However, the evidence is lacking [14-16] on the development and evaluation of apps for their use in routine clinical care. To guide the development of further apps for patient self-management, it is essential to gain insight into the perceptions and experiences of patients living with long-term conditions, such as RMDs, who are current or potential users of available apps. Through a mixed-methods approach, this study explores the needs, experiences, and views of people with RMDs about their knowledge and use of mHealth apps. The aim was to obtain direct patient feedback on preferences for content, functionalities, structure, navigation, and relevance for their long-term condition and an evaluation of mHealth apps for self-management of their RMDs.

## Methods

### Overview

This was a mixed-methods study starting with one patient focus group to gather direct patient insights on the subject. An online, multinational survey followed, informed by the themes emerging from the focus group in the UK.

### Patient Focus Group

One audio-recorded patient focus group in the UK was facilitated by an experienced qualitative researcher (HL) in March 2018; a cofacilitator was also present and took notes during the focus group. Patient research partners affiliated with the department of rheumatology at King’s College London were invited by email. After they agreed to participate, an information sheet (developed by AN and EN) was sent to all to explain the purpose of the study.

Broad and open-ended questions were developed for the semistructured focus group guide along with specific questions about the use of mobile health apps for the management of their RMDs (see focus group schedule in Multimedia Appendix 1). The focus group lasted 75 minutes and took place in a private room in a medical school building. The analysis was performed through an inductive approach, carrying out three coding phases and identifying emerging key themes.

### Online Open Survey

Key themes that emerged from the patient focus group informed the design of the next phase, namely the patient survey to obtain further detailed information on the subject of mHealth apps. The survey was created by a panel of four rheumatologists (AN, EN, LG, FB). A draft survey was shared with five patient research partners who live with RMDs from across five different countries (UK, Slovakia, Germany, the Netherlands, Cyprus) for direct feedback on the structure, relevance, and comprehension of the content, validation with pretest, and appropriate rephrasing when needed before its finalization and dissemination. The results are reported according to the CHERRIES (Checklist for Reporting Results of Internet E-Surveys) checklist [17]. Consent was collected before entering the study. The final survey contained 40 questions, divided into five main sections: (1) sociodemographic information about the patients, (2) current mobile phone use, (3) current knowledge and use of apps for RMDs, (4) past use of apps for RMDs, and (5) development of the ideal app for self-management. Most questions were multiple choice, with a few open-ended ones.

### Survey Dissemination

For dissemination of the survey, the sample frame was defined as people living with RMDs. Individual patient organizations from 45 EU countries, the United States, Canada, and Australia were approached.

For each EU country, dedicated People with Arthritis and Rheumatism (PARE) representatives linked to each European League Against Rheumatism (EULAR) country were contacted directly via email by the study team. Each country representative was responsible for contacting registered patient associations. Patient organizations then disseminated the survey through their websites and social media channels (Twitter). In the countries not linked to EULAR, patient associations or scientific societies were contacted to support the dissemination process.

The survey was translated in Slovakian, Portuguese, Spanish, and French as requested by national patient research partners in charge of dissemination. The survey dissemination was conducted in 2018. Informed consent was collected at the beginning of the survey and was mandatory to start the survey; participants were informed of the expected duration of the survey, purpose, and data storage modalities. The survey contained 41 items on nine pages. It was made available for completion from mid-May to late June through Survey Monkey.

### Data Analysis

The information provided by participants during the focus group was transcribed verbatim by AN. The data were thematically analyzed manually [18] by one researcher and one rheumatologist in the research team (AN, EN). A further cross-check of the emerging themes was carried out by HL and a patient involved in the focus group; single counting was applied (number of events, phenomena identified during the analysis) [19]. Final agreement among the research team (AN, EN, LG, FB) was reached for the key themes to be presented within an analytical framework. The survey results were collected and analyzed by AN through descriptive statistics using Excel (Microsoft Office) and Graphpad software [20].

## Results

### Patient Focus Group

Three of six focus group participants were female, with an age range between 32 and 69 years, and backgrounds of diverse disease duration and level of disability related to their disease (Multimedia Appendix 1). All attended the same tertiary outpatient clinic in London and lived in different parts of England. All had a diagnosis of a long-term autoimmune inflammatory RMD condition (five had rheumatoid arthritis [RA], one had myositis). Disease duration ranged from 3 to 47 years. All were medically retired, meaning the participants left their jobs before they had reached the official retirement age due to a health condition or sickness. They had diverse ethnic and socioeconomic backgrounds.

#### Summary of Key Emerging Themes

The analytical framework consisted of five key themes: (1) knowledge and previous use of mHealth apps in general, (2) experience of mHealth apps for self-management in rheumatology, (3) positive and negative features of the apps, (4) need for improvement in self-management apps, and (5) content and functionalities of ideal self-management apps for RMDs.

#### Knowledge and Previous Use of mHealth Apps in General

Three of six patients had heard of the term *eHealth* at least once and knew what the phrase referred to. eHealth was defined as “an emerging field in the intersection of medical informatics, public health, and business, referring to health services and information delivered or enhanced through the internet and related technologies*”* [21]:

I remember when I talked to my consultant, and he let me know that there was an app apparently that you could go into and basically put all your appointments.

patient 1, female

I would find this [app] useful if you want to know where to go in the hospital, and it has phone numbers from the departments.

patient 2, male

Most patients (4/6) were aware of the actual existence or the current development of apps for patients with RMDs: “My consultant told me about [the developments of apps]” (patient 2, male).

Two of six patients used general health apps (not designed specifically for patients living with RMDs), such as activity trackers or pedometers. Activity trackers and pedometers were defined as devices that use sensors to help users automatically track step counts while aiming for a particular step count or activity goal. Pedometers help promote self-awareness and self-monitoring of activity levels [22]. Such devices were perceived positively by the group: “It [addition of an app] makes me more conscious of the steps I do” (patient 4, female).

Half of the informants (3/6) reported that the pedometers could also be discouraging:

I had the feeling to do thousands of steps, and it [pedometer] was telling me not [participant had the impression of having done a lot of activity; however, the pedometer displayed a low number of counted steps]. It was actually demotivating, and I put it in the bin.

patient 1, female

#### Experience of mHealth Apps for Self-Management in Rheumatology

A third of patients had used mHealth apps for their rheumatic disease:

I have used it [app], I felt it was pretty time-consuming as you had to put so much in information) it [app], especially for the blood test.

patient 6, male

I am aware of a couple [of apps] through the NRAS [National Rheumatoid Arthritis Society, National Charity UK], the DAS [Disease Activity Score] app ...I also was involved in trying “Rheumopady,” it is an app where you daily mark [input] your fatigue, sleep, pain, and stiffness, and it [app] marks a graph for you.

patient 5, female

Most (5/6) expressed enthusiasm about the incorporation of apps for self-management into their daily lives:

That is why I think it is app] very relevant, because I usually write these [symptoms] in a diary, how I feel.

patient 2, male

A graph would be great to show trends...a graph is a simple image you could show to the consultant.

patient 4, female

I think it [app] would be useful for people who are still working to see...because often they [clinicians, friends, families] don’t realize how much time people have pain, even though they [patients] seem fine all day, they [patients] are probably not well-controlled.

patient 5, female

One patient voiced reluctance to use an app due to lack of knowledge about the function and therefore decided not to buy one:

“I was never going to put bloods [tests] into it [app] because, you know, the people who need to know got the info in the medical notes, I don’t look at mine [blood tests] anyway”.

patient 1, female

None of the participants were using bespoke RA self-management apps at the time of the interview.

#### Positive and Negative Features of the Apps

Positive and negative features were discussed by the participants, as well as the pros and cons of using self-management apps. Some participants (2/6) reported that RA self-management apps were helpful in principle, such as to remind them of their disease progress and their symptoms over time (eg, between clinic appointments). The majority (5/6) mentioned that apps could help them to remember and describe their symptoms more accurately to the clinicians as a “symptom diary”:

I think it depends what [the information on the app] is going to be used for, because I think sometimes it would be useful, to put flares in [the app], because it seems so far, we are always fine on the day we go to clinics.

patient 5, female

I think it depends on the quantity of information asked [to be included in the app]...it was easy to type from 0-10 [for the VAS] for different health parameters.

patient 4, female

However, others (3/6) stated that self-management apps tend to take too much time to enter data every day and were rarely accessible or easy to use:

It’s too much hassle, I mean, it was interesting, but I found it [app] was such a *** some days, it would save the data for you and some days it wouldn’t. It was a trial [used the app during a trial], but if it was in real life I would not have carried on. If you put things [data] in that [app], fine, that’s great, the principle is amazing. I think it is really interesting, you could see the graphs...but the fact is that it [app] did not work as well as it should have...The functionality is a big issue.

patient 4, female

It [the process] was so time-consuming...taking five minutes to go online.

patient 3, male

That kind of apps frustrate me generally; I don’t find that they [apps] usually work as much as they should.

patient 5, female

#### Need for Improvement in Self-Management Apps

Participants (2/6) expressed a need for an improvement in the intuitive functionality of apps, especially regarding the time needed for data and information entry into the app.

An app allowing data collection and description of the symptoms would facilitate more relevant outpatient visits as patients tend not to remember how they have been feeling or experiencing symptoms over the past few weeks:

Why I think is very relevant, is because every time I go to see the consultant...and they ask you about not only how you be feeling over the last 7 days, which maybe you could remember...but then you maybe miss the kind of patterns, especially over months...and then that is not always indicative.

patient 2, male

It has to be easy too, for the older people, very often you hear this in clinics: “I can’t do that.”

patient 1, female

#### Content and Functionalities of Ideal Self-Management Apps for Rheumatic and Musculoskeletal Diseases

According to most focus group participants (4/6), the key domains to be addressed by an “ideal” app should include the following features: assessment and measurements of fatigue, sleep, pain, mood, activity, symptoms, Disease Activity Score (DAS 28), and appetite. All patients (6/6) suggested that such features in an app could help them in their self-management of their long-term conditions within the context of their daily activities (eg, planning social events, traveling) (Textbox 1). Moreover, they discussed features, functionalities, and potential links for inclusion into an “ideal app” as detailed in Textbox 1: “It would be quite nice to have a link with explanations on why you are taking this drug” (patient 6, male).

**Domains suggested by patients for how an app could be helpful in the self-management of their rheumatic and musculoskeletal diseases, including special features that result in an “ideal app.”**

**Features**

- The ability to analyze and draw correlations between symptoms (eg, fatigue, pain, activities), blood tests, medication, and disease activity
- The possibility of identifying specific “patterns” of symptom features that could predict flares
- Display graphs and trends of the inputted data/info
- The ability to quantify and share fatigue levels with the rheumatologist
- Information on medication adherence (reminders and other information about the importance of medication adherence)
- Blood test and outpatient clinic appointment reminders
- Records of blood monitoring results

**Ease of use**

- Availability on a tablet (easier to navigate with a bigger screen, easy use of keyboard with painful fingers due to RMD)
- Ease of use (intuitive functionality) especially in the case of “older people”

**Disease monitoring (self-management)**

- Therapeutic and management advice, subject to health parameters (eg, high disease activity score=advice to increase steroids or other treatment)
- Visual personalized health information through graphs and trends

**Useful information and website links**

- Information about medication side effects and interactions
- Links to websites to explain the purpose of each medication
- Links to national organization/helpline (national RMDs Society or Charity)
- Explanations and advice about the disease and some aspects of lifestyle changes (eg arthritis and fatigue, arthritis and sex)

**Reminders (self-management)**

- Option to set reminders to enter health parameters (eg, fatigue, sleep, pain, mood, activity, symptoms, DAS 28, blood tests)
- Notifications/alerts (eg, when new medication supply is needed)

### Online Open Survey

#### General Demographics

Among the 429 respondents, 394 (91.8%) participants provided complete responses (all mandatory questions completed). The majority (86.9%, 342/394) of respondents were female, and almost half of the participants were 18 to 44 years of age. Sociodemographic information and diagnoses of the participants are detailed in Table 1.

Country representation varied, with the highest number of responses from Portugal (84/394, 21.3%), followed by Germany (50/394, 15%), Australia (33/394, 8.4%), United States (36/394, 9.1%), and Cyprus (25/394, 6.3%). Other participating countries are shown in Multimedia Appendix 2.

**Table 1:** Demographics and disease-related information of participants (N=429).

Demographic		n (%)
**Age range (years)**		
	18-24	15 (3.8)
	25-34	50 (12.7)
	35-44	98 (24.9)
	45-54	113 (28.7)
	55-64	87 (22.1)
	65 and older	31 (7.9)
**Gender**		
	Female	342 (86.8)
	Male	50 (12.7)
	Transgender male	1 (0.3)
**Disease^a^**		
	Rheumatoid arthritis	152 (38.8)
	Fibromyalgia	80 (20.4)
	Psoriatic arthritis	76 (19.4)
	Ankylosing spondylitis or spondyloarthritis	67 (17.1)
	Osteoarthritis	64 (16.3)
	Sjögren syndrome	55 (14.0)
	Spine or back disease	36 (9.2)
	Systemic lupus erythematosus	31 (7.9)
	Juvenile idiopathic arthritis	30 (7.7)
	Osteoporosis	29 (7.4)
	Vasculitis	11 (2.8)
	Scleroderma or systemic sclerosis	14 (3.6)
	Polymyositis/dermatomyositis	4 (1.0)
	Other	65 (16.6)

^a^The participant percentage total is greater than 100% because multiple choices were possible.

Patients were diagnosed mostly with RA (152/394, 38.6%), fibromyalgia (80/394, 20.3%), psoriatic arthritis (76/394, 19.3%), and ankylosing spondylitis (67/394, 17%). Almost half of the patients (191/394, 48.5%) had their main diagnosis for more than 10 years.

### Current and Past Use of Mobile Phone and mHealth Apps for Rheumatic and Musculoskeletal Diseases

Most patients used a mobile phone (355/394, 90.1%), mostly for text messages and calls (341/355, 95.1%) or internet access (328/355, 92.4%) and social media use (eg, Facebook, Twitter, Instagram, Snapchat; 289/355, 81.4%) (Table 2). A majority (341/358, 95.3%) reported searching for information on the internet about their disease. Half of the respondents were aware of the existence of apps to support them in self-managing RMDs (188/355, 52.3%). Among them, almost half of the respondents were currently using a self-management app (79/188, 42%), a third of them on a weekly basis (Table 2).

**Table 2:** Current and past use of mobile phone apps for rheumatic and musculoskeletal diseases (N=358).

Use of mobile phone apps^a^		n (%)
**Purposes for the use of a mobile phone^b^** **(n=358)**		
	Text messages and calls	341 (95.3)
	Internet access	328 (91.6)
	Games	115 (32.1)
	Social networking sites (eg, Facebook, Twitter, Snapchat, Instagram)	289 (80.78)
	Other apps	196 (54.7)
**Knowledge about mHealth apps for self-management of RMDs (n=355)**		
	Yes	188 (53.0)
	No	167 (47.0)
**Current use of an app for RMD self-management (n=188)**		
	Yes	79 (42.0)
	No	109 (58.0)
**Frequency of use of an app for RMD self-management (n=67)**		
	On a daily basis	20 (29.9)
	On a weekly basis	21 (31.3)
	On a monthly basis	8 (11.9)
	Less than once monthly	3 (4.6)
	Intermittently	15 (22.4)
**Past use of an app (n=106)**		
	Yes	44 (41.5)
	No	62 (58.5)
**Duration of use (n=41)**		
	Less than 3 months	31 (75.6)
	3-6 months	8 (19.5)
	More than 6 months	2 (4.9)
**Reason for discontinuing use^b^** **(n=37)**		
	I did not find it/them useful for my condition	24 (64.9)
	I feel it did not benefit my overall health	16 (43.2)
	I found it/them too time-consuming	15 (40.5)
	I got bored	11 (29.7)
	I did not like the design or user interface	10 (27.0)

^a^The n in parentheses represent the total number of participants who answered each question

^b^Total is greater than 100% because multiple choices were possible.

The reported app usage was mainly for disease management (42/67, 62.7%) and coping with arthritis-related symptoms and consequences (28/67, 41.8%) (Multimedia Appendix 2). Medication intake monitoring (24/67, 35.8%) was the third most common reported use.

The patients received recommendations about the apps by patient associations (29/67, 43.3%), advertisements (eg, print media, online social media; 24/67, 35.8%), and friends or relatives (13/67, 19.4%). Apps were recommended by physicians or health care professionals only in a few cases (10/67, 14.9%).

Almost half of the nonusers described a previous trial of a self-management app for less than three months. They mostly stopped using it because they did not find them helpful (24/37, 64.9%), saw no benefit for their health (16/37, 43.2%), found the device too time-consuming (15/37, 40%), got bored of using the app (11/37, 29.7%), or they did not like the design or user interface (10/37, 27%).

For respondents who declared no usage of apps for disease management, the reasons provided were the following: no benefit for their health (33/105, 31.4%), fear they would spend too much time on filling the app (27/105, 25.7%), or concerns about health data protection (25/105, 23.8%) (Multimedia Appendix 3).

### Features and Functionalities of Ideal Self-Management Apps for Rheumatic and Musculoskeletal Diseases

The majority of patients were interested in an app that helped with self-monitoring of health parameters (259/346, 74.9%), disease activity (221/346, 63.9%), communication with their health care providers (221/346, 57.8%), and information about their disease (200/346, 53.5%). Multimedia Appendix 4 details other reported features and functionalities of “ideal” self-management apps for RMDs. The collection of anonymized health data for research purposes seemed acceptable by most patients (n=176/320, 57.9%), as shown in Multimedia Appendix 5. The aspects to be addressed and information to be collected by the ideal app are described in Table 3.

**Table 3:** Features to be collected by the ideal App (N=345).

Features	n (%)
*Pain* ^a^	*186 (84.2)*
*Fatigue*	*153 (69.2)*
*Physical Activity*	*149 (67.4)*
Sleep	118 (53.4)
Disease activity score	118 (53.4)
Well-being	119 (53.8)
Medication, adherence	111 (50.2)
Morning stiffness	112 (50.7)
Blood tests	112 (50.7)
Nutrition	106 (47.9)
Depression/Anxiety	97 (43.9)
Infections	95 (42.9)
Mood	86 (38.9)
Social support	70 (31.7)
Fears	61 (27.6)
Work	64 (28.9)
Other^b^	15 (6.8)

^a^Top responses are highlighted in italic.

^b^Other: Included Flares, Heart Rate, Blood Pressure, Temperature, a place to keep track of how the weather impacts pain level, and X-ray results.

Patients suggested they would like the information collected by the app to be shared with their rheumatologist (277/345, 80.1%), their general practitioner (240/345, 69.4%), and health professionals (eg, physiotherapist, nurse; 254/345, 73.4%). A majority (325/346, 93.9%) stated that they prefer to have control over which health care professionals have access to their personal data through the app because this could make clinic visits more efficient (322/346, 93.1%). Interesting concepts emerged related to either self-management strategies (eg, pain, fatigue, physical activity) or other aspects such as work and social support.

The majority of respondents (197/338, 58.3%) expressed an unwillingness to pay for an app. From those prepared to pay, most (85/141, 60.3%) expressed their preference for a one-off payment (Multimedia Appendix 6).

Patients expressed highest confidence in an app developed by an RMD scientific society (very confident: 207/331, 62.5%). Poorest confidence was expressed for an app developed by a pharmaceutical company (152/331, 45.9%).

Regarding the operating mode of the app, the majority (280/320, 87.5%) were in favor of the app to send reminders, with half of them in favor of receiving reminders for medication intake (149/280, 53.2%) and health appointments (162/280, 57.8%). Most respondents (295/320, 92.2%) were keen to include a display of visual health parameters with images (95/320, 29.7%), tables (71/320, 22.2%), or graphics (129/320, 40.3%).

A majority (306/320, 95.6%) of the respondents wanted to be given the opportunity to rate the app: 18.4% (59/320) wanted to do so by voting, 50.9% (163/320) by using a satisfaction scale, and 22.8% (73/320) by providing free-text comments.

## Discussion

This study assessed patients’ perceptions of and experiences with mHealth apps for self-management in rheumatology. It also explored people’s views on what they perceive as an ideal app. Our study highlights the willingness of people with RMDs to use such apps to improve their disease management. The latter could potentially be through patient empowerment and encouraging patients to take a more active role in decisions relating to their health. Our study also highlights the need for improvement in app functionalities and content, a desire that was consistently expressed across both the patient focus group and the survey.

Despite the recent rapid growth in the development and dissemination of apps for disease self-management, our focus group and survey results demonstrated that half the respondents did not know about the existence of mHealth apps in rheumatology. This might be explained in part by the wide range of ages represented by our survey participants, with 58.7% older than 45 years (231/394). These results are in line with another recent survey on cancer apps, in which the age of the patient (age range 18-39 years) was significantly correlated with the use of the app [23], although the correlation was weak. However, a recent survey performed with type 1 diabetes patients, who represent a younger patient population by definition, showed that 61% of the patients reported no awareness or knowledge of the existence of self-management apps [24]. These observations may indicate that other reasons may be at play that are not related to age, such as lack of health care provider awareness or willingness to encourage app use or directly related to the underlying condition (eg, joint swelling and deformities in rheumatic diseases preventing easy use of apps). The focus group revealed the wish of patients for apps to be tailored for use by “older patients.” From a financial point of view, patients expressed their preferences for a free app.

Interestingly, patients who had used available mHealth apps for RMD self-management had an overall negative opinion, and most stopped using them for various reasons (eg, too time-consuming to log in or to complete their health parameters and with no direct positive impact on their health). For example, patients mentioned they were more likely to use an app when they were feeling well and when not undergoing a flare. Moreover, poor functionality of certain apps rendered them unreliable, which caused frustration. These findings are in line with a similar survey conducted in other medical specialties (eg, in type 1 diabetes [24]). Patients did not think the app benefited their health because data, especially information on glycemia across the day, could not be shared with their doctor. The lack of immediate feedback on the blood results and without therapeutic action was perceived as unsatisfactory. Indeed, the creation of apps designed for people living with RMDs represents a relatively recent change [25]. Previous work that assessed the quality of existing apps in Google Play, iTunes, or Android app stores showed that most of the available ones did not meet high quality standards [26-28]. Moreover, based on a previous systematic literature review from our group [29], the majority of apps and devices for patients living with RMDs were not developed with any input from patients. This is a major drawback. This oversight of the direct inclusion of relevant stakeholders might lead to irrelevant and inappropriate development processes.

Data sharing with health care providers does raise ethical issues [30,31]. Based on our survey, health data protection was also a reason that was frequently stated for not using mHealth apps (25/105, 23.8%). A majority of patients from both our multinational survey and focus group expressed a wish to be able to choose if and which health care providers could access their health data. Previous work has proposed or summarized existing regulations on the safety and security of mobile apps [32-35]. Moreover, apps and any other e-device should follow the EU General Data Protection Regulation (GDPR) and US Health Insurance Portability and Accountability Act (HIPAA). However, to our knowledge, no specific regulation on which health care providers should access the data gathered by the apps exists yet.

Our study specifically raised questions about the content and functionalities of apps that people with RMDs wished to see in an ideal app. A broad range of features was described, such as pain, fatigue, physical activity, sleep, and morning stiffness. However, many of these details were not yet included or only partially included in existing apps currently available on the market [30,31]. We acknowledge the challenges of developing an app that is inclusive of all functions desired by study participants also from a technical point of view. Furthermore, we acknowledge that people with RMDs can have significant limitations in their function, including fine movement of the hands and fingers, for example, which poses practical challenges in the use of apps. The app needs to be developed to support and encourage a proactive role of patients in the management of their health. The elaboration of a single app might not necessarily be able to provide all described features. A good way to make the app straightforward and practical is to involve patients in the development process.

Therefore, our results are important to provide further guidance on how best to tailor newly developed apps to patient needs in the field of RMDs.

Our study provides an in-depth approach to studying views, needs, preferences, and previous experiences of mHealth apps in patients living with RMDs. Using a mixed-methods approach enabled the research team to identify key aspects of mHealth app features and functionalities that have the potential to positively influence future development of apps. Moreover, many of the emerging themes were in line with the five core self-management skills (pain education, self-efficacy building, self-monitoring, social support, and goal setting) [36].

Our online, multinational survey, translated into five different languages, made it possible to reach out to the wider patient community with RMDs and to obtain first-hand patient feedback. The survey was disseminated and completed by participants with a broad range of age categories, diseases, and countries across three different continents. Limitations of our study include the single focus group performed in one country. In addition, because of the qualitative arm of the study results and that the online survey was open to everyone, the results might not necessarily be generalizable. Furthermore, most of the participants in the focus group had a diagnosis of RA, which may affect the generalizability of the results to other RMDs.

However, our survey provided greater granularity of information on specific themes that emerged from the focus group, as well as more generic themes confirmed in the systematic review of the literature and expert opinion. The higher number of female patients participating in the survey is in line with existing epidemiological data, which supports that the gender ratio is in favor of women in highly prevalent RMDs, such as fibromyalgia and RA [37,38].

In conclusion, despite the reluctance of some people with RMDs to incorporate apps for reasons such as relevance of content, reliable functionality, and data safety concerns, there was acknowledgment that apps may be suitable for patient self-management. If apps are bespoke and developed in close collaboration with the key stakeholders (ie, the patients and carers), this inclusive approach may have potential benefits for patient health. The development of such apps will require standardization and quality control processes to be in place [16]. There is an unmet need to develop helpful and tailored mHealth apps. It is essential that patients can be sure of the accuracy and safety of their personal data, including sociodemographic data. National RMD societies and patient associations have an important role to play in the development, validation, and dissemination of mHealth apps among patients and health care providers.

## Appendix

Multimedia Appendix 1 Patient characteristics.

Multimedia Appendix 2 Demographic data: country of origin of the survey participants.

Multimedia Appendix 3 Reasons for app use reported by participants.

Multimedia Appendix 4 Reasons for not using apps for RMDs.

Multimedia Appendix 5 Purposes of the ideal app.

Multimedia Appendix 6 Patients willingness to pay for the app.

## Abbreviations

DAS: Disease Activity Score
EULAR: European League Against Rheumatism
RA: rheumatoid arthritis
RMD: rheumatic and musculoskeletal diseases
