# Supplementary material for: Needs, Experiences, and Views of People With Rheumatic and Musculoskeletal Diseases on Self-Management Mobile Health Apps: Mixed Methods Study
Source: JMIR Mhealth Uhealth. 2020 Apr 20;8(4):e14351. doi: 10.2196/14351 (PMC7199138; doi:10.2196/14351)
Supplement: Multimedia Appendix 1 [file mhealth_v8i4e14351_app1.docx]

Supplementary Table 1

Table 1. Patient characteristics.

| **Patient** | **Gender** | **Disease** | **Age** | **Disease duration** | **Ethnicity** |
| --- | --- | --- | --- | --- | --- |
| Patient 1 | Female | RA | 69 | >25 yrs. | English |
| Patient 2 | Male | RA | 56 | 7 years | Black-European |
| Patient 3 | Male | RA | 48 | 3 years | English |
| Patient 4 | Female | RA | 32 | 12 years | Welsh |
| Patient 5 | Female | RA | 50 | 47 years | English |
| Patient 6 | Male | Myositis | 64 | 13 years | English |
