# Supplementary material for: Needs, Experiences, and Views of People With Rheumatic and Musculoskeletal Diseases on Self-Management Mobile Health Apps: Mixed Methods Study
Source: JMIR Mhealth Uhealth. 2020 Apr 20;8(4):e14351. doi: 10.2196/14351 (PMC7199138; doi:10.2196/14351)
Supplement: Multimedia Appendix 2 [file mhealth_v8i4e14351_app2.doc]

*Demographic data: country of origin of the survey participants.*

| **Country** | **Number** | **%** |
| --- | --- | --- |
| Portugal | 84 | 21.3 |
| Germany | 59 | 15.0 |
| United States of America | 36 | 9.1 |
| Australia | 33 | 8.4 |
| Cyprus | 25 | 6.3 |
| United Kingdom | 22 | 5.6 |
| France | 21 | 5.3 |
| Czech Republic | 16 | 4.1 |
| Canada | 13 | 3.3 |
| Slovakia | 13 | 3.3 |
| Spain | 12 | 3.0 |
| Netherlands | 9 | 2.3 |
| Romania | 8 | 2.0 |
| Serbia | 8 | 2.0 |
| Italy | 5 | 1.3 |
| Belgium | 3 | 0.8 |
| Bulgaria | 2 | 0.5 |
| Denmark | 2 | 0.5 |
| Estonia | 2 | 0.5 |
| Iceland | 2 | 0.5 |
| Ireland | 2 | 0.5 |
| Greece | 1 | 0.3 |
| Hungary | 1 | 0.3 |
| Lithuania | 1 | 0.3 |
| Luxembourg | 1 | 0.3 |
| Malta | 1 | 0.3 |
| New Zealand | 1 | 0.3 |
| Poland | 1 | 0.3 |
| Russian Federation | 1 | 0.3 |
| Slovenia | 1 | 0.3 |
| Switzerland | 1 | 0.3 |
| Turkey | 1 | 0.3 |
