# Supplementary material for: Needs, Experiences, and Views of People With Rheumatic and Musculoskeletal Diseases on Self-Management Mobile Health Apps: Mixed Methods Study
Source: JMIR Mhealth Uhealth. 2020 Apr 20;8(4):e14351. doi: 10.2196/14351 (PMC7199138; doi:10.2196/14351)
Supplement: Multimedia Appendix 3 [file mhealth_v8i4e14351_app3.doc]

*Reasons for App use reported by participants.*

| Purpose of the App | N | % |
| --- | --- | --- |
| *Disease-management* | *42* | *62.7* |
| *Coping with arthritis related symptoms and consequences* | *28* | *41.8* |
| *Medication intake monitoring* | *24* | *35.8* |
| Physical Activity Monitoring | 20 | 29.9 |
| Dealing with side effects | 20 | 29.9 |
| Self-rehabilitation | 19 | 28.4 |
| Help with treatment adherence | 18 | 26.9 |
| Communication with physicians | 17 | 25.4 |
| Communication with other patients | 17 | 25.4 |
| Other | 5 | 7.5 |
| Total | 67 |  |

*Top responses are highlighted in italic.*

* Other included: relaxation exercises, obtain information about the disease.
