# Supplementary material for: Needs, Experiences, and Views of People With Rheumatic and Musculoskeletal Diseases on Self-Management Mobile Health Apps: Mixed Methods Study
Source: JMIR Mhealth Uhealth. 2020 Apr 20;8(4):e14351. doi: 10.2196/14351 (PMC7199138; doi:10.2196/14351)
Supplement: Multimedia Appendix 4 [file mhealth_v8i4e14351_app4.doc]

*Reasons for not using Apps for RMDs.*

|  | **N** | **%** |
| --- | --- | --- |
| *It does not benefit my health* | *33* | *31.4* |
| *It takes up too much of my time* | *27* | *25.7* |
| *Concerns relating to health data protection* | *25* | *23.8* |
| *I am not willing to pay* | *22* | *21.0* |
| Storage space on my smartphone or tablet | 19 | 18.1 |
| Not necessary for my condition | 19 | 18.1 |
| The app requires Wi-Fi or cellular data which I don’t have constant access to | 14 | 13.3 |
| I dislike the design / user interface | 14 | 13.3 |
| There is no suitable app in my native language | 13 | 12.4 |
| I feel it is useless | 11 | 10.5 |
| No trust in the content | 8 | 7.6 |
| Most apps are not compatible with the operating system of my smartphone or tablet | 7 | 6.7 |
| No trust in the app developer (e.g. it isn’t available on the App store or on Google Play) | 7 | 6.7 |
| I was not aware of such apps | 7 | 6.7 |
| Not familiar with using a smartphone or tablet | 1 | 1.0 |
| No smartphone or tablet | 0 | 0.0 |
| Total | 105 |  |

*Top responses are highlighted in italic.*
