# Supplementary material for: Needs, Experiences, and Views of People With Rheumatic and Musculoskeletal Diseases on Self-Management Mobile Health Apps: Mixed Methods Study
Source: JMIR Mhealth Uhealth. 2020 Apr 20;8(4):e14351. doi: 10.2196/14351 (PMC7199138; doi:10.2196/14351)
Supplement: Multimedia Appendix 5 [file mhealth_v8i4e14351_app5.docx]

*Purposes of the ideal App.*

| Purposes of the ideal App | N | % |
| --- | --- | --- |
| *Self-monitoring of your health (e.g pain. fatigue. physical activity…)* | *259* | *74.9* |
| *Monitoring of disease activity* | *221* | *63.9* |
| *Communication with physicians and other health professionals* | *200* | *57.8* |
| *Information about the disease* | *185* | *53.5* |
| Coping with arthritis-related symptoms and consequences | 183 | 52.9 |
| Dealing with side effects of medication | 177 | 51.2 |
| Monitoring lifestyle (physical activity. calorie intake. smoking) | 167 | 48.3 |
| A place to store my medical records | 155 | 44.8 |
| Managing medication-intake | 153 | 44.2 |
| Booking and managing appointments | 138 | 39.9 |
| Communication with other patients | 130 | 37.6 |
| Information about healthcare and medical things in general | 130 | 37.6 |
| Ordering prescriptions | 120 | 34.7 |
| Other (please specify) | 22 | 6.4 |
| Playing games with other patients | 20 | 5.8 |
| Total | 346 |  |

*Top responses are highlighted in italic.*

* Other included:
