# Supplementary material for: Needs, Experiences, and Views of People With Rheumatic and Musculoskeletal Diseases on Self-Management Mobile Health Apps: Mixed Methods Study
Source: JMIR Mhealth Uhealth. 2020 Apr 20;8(4):e14351. doi: 10.2196/14351 (PMC7199138; doi:10.2196/14351)
Supplement: Multimedia Appendix 6 [file mhealth_v8i4e14351_app6.docx]

*Patients willingness to pay for the App.*

| Willingness to pay for the App | N | % |
| --- | --- | --- |
| Yes | 141 | 41.7 |
| *No* | *197* | *58.3* |
| Type of payment |  |  |
| *A one-off payment* | *85* | *60.3* |
| A monthly payment | 27 | 19.4 |
| A yearly payment | 21 | 15.1 |
| Other* | 6 | 4.3 |
| Amount (total) |  |  |
| Less than 1€/$ | 5 | 3.6 |
| 1-2€ | 17 | 12.3 |
| *2-5€* | *71* | *51.4* |
| More than 5€ | 45 | 32.6 |
| Total | 338 |  |

*Top responses are highlighted in italic.*

* Other included: It Depends on how much and how the money generated would be used.
